# P-Selectin is a Critical Factor for Platelet-Mediated Protection on Restraint Stress-Induced Gastrointestinal Injury in Mice

**DOI:** 10.3390/ijms231911909

**Published:** 2022-10-07

**Authors:** Subhashree Pethaperumal, Shih-Che Hung, Te-Sheng Lien, Der-Shan Sun, Hsin-Hou Chang

**Affiliations:** 1 Department of Molecular Biology and Human Genetics, Tzu-Chi University, Hualien 970, Taiwan; 2 Institute of Medical Sciences, Tzu-Chi University, Hualien 970, Taiwan

**Keywords:** platelet transfer, restraint stress, gastrointestinal injury, gastrointestinal leakage, gastrointestinal epithelial cell, apoptosis, P-selectin

## Abstract

Psychological stress is associated with increased risk of gastrointestinal (GI) tract diseases. Evidence indicated that platelets facilitate GI tissue repair in intestinal anastomosis models. However, whether platelets are involved in native mechanism of the rescue of stress-induced GI injury for maintaining the GI homeostasis remains elusive. Because P-selectin-deficient (*Selp^−/^^−^*) mice displayed higher stress-induced GI injury compared to the wild-type (*Selp^+/+^*) mice, and P-selectin is specifically expressed in platelets, we hypothesize that P-selectin-expressing platelets play a protective role in the rescue of stress-induced GI injury. Our goal is to clarify the putative protective role of platelets in a GI system, thereby develop a feasible intervention strategy, such as platelet transfer, to overcome stress-induced GI injury. Through monitoring the plasma levels of GI-nonabsorbable Evans blue dye to reveal the progression course of GI injury in live mice, we found that intravenous treatments of purified platelets ameliorated stress-induced GI leakage. The transfer of platelets from wild-type mice was more potent than from *Selp^−/^^−^* mice in the rescue of stress-induced-GI leakage in the recipients. As such, platelet transfer-mediated rescue was conducted in a P-selectin dependent manner. Additionally, platelet-mediated protection is associated with corrections of stress-induced aberrant GI mRNA expressions, including tight junctions claudin 3 and occludin, as well as stress-induced genes activating transcription factor 3 and AMP-activated protein kinase, after the transfer of wild-type platelets into wild-type and *Selp^−/^^−^* mice. Furthermore, the stress-induced apoptosis of CD326^+^ GI epithelial cells was rescued by the transfer of wild type, but not P-selectin-deficient platelets. These results suggest that platelet plays a protective role for maintaining the GI homeostasis during stress in vivo, and that P-selectin is a molecular target for managing stress-induced GI tract injury.

## 1. Introduction

The risk of gastrointestinal (GI) tract diseases is associated with mental stress [1,2,3]. For instance, an increased risk of GI tract diseases is linked to major psychological disorders [4], such as depression and anxiety [2], schizophrenia [5], bipolar disorder [6], dementia [7,8], and autism [9]. The bidirectional communication of the gut–brain axis, which consists of the central nervous system, as well as neuroendocrine, immune and GI systems [10,11], partly explains the associations between psychological stress and GI tract disorders. However, the mechanism underlying the induction and reversal of mental stress-induced GI injuries remains elusive.

A mouse model of restraint stress is a well-recognized tool to study mental stress–related physiological, behavioral, and biochemical changes in mice [12,13,14]. Pathophysiological changes associated with anxiety and stressed behavior of experimental animals could occur after restraint stress [12]. Evans blue, a GI-nonabsorbable dye, will not normally appear in plasma when orally fed [15]. By examining the plasma levels of Evans blue, we can measure stress-induced GI leakage in real time [15]. In our previous study that used Evans blue and restraint stress mouse model, acute restraint stress leads to GI leakage, which is associated with the death of gut epithelial cells [15]. However, the detailed mechanism remains unclear.

Upon activation by injuries, platelets express adhesion receptor P-selectin [16], a membrane protein stored in platelet alpha-granules, which binds to P-selectin glycoprotein ligand-1 (PSGL-1) on leukocytes and cells in injured tissues [17]. P-selectin is specifically expressed by platelets and endothelial cells [16,18]. In the present study, we found that the expression levels of platelet surface P-selectin are markedly up-regulated after restraint stress. Given the evidence that P-selectin [19,20,21] and platelets [17] exert protective roles during injuries, we hypothesize that P-selectin expressing platelets may have a protective role in the suppression of stress-induced GI injury. Results indicated that P-selectin gene deficient (*Selp^−/^^−^*) mice displayed more severe GI leakage than wild-type (*Selp^+/+^*) mice, suggesting that P-selectin ameliorates GI injuries. In addition, platelets transferred from wild type, but not P-selectin-deficient mice ameliorated the stress-induced GI leakage. Hence, platelets exert GI rescuing property through the P-selectin pathway.

## 2. Results

### 2.1. Involvement of P-Selectin^+^ Platelets in GI Protection during Stress-Induced GI Injury

**Figure 1:**
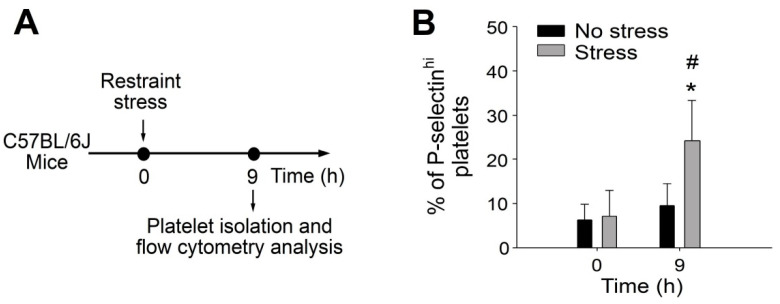
Percentage of circulating P-selectin^hi^ platelets was up-regulated after restraint stress in mice. (**A**) Experiment outline of restraint stress mouse model and platelet analysis. (**B**) Percentage of platelets expressing high surface P-selectin (P-selectin^hi^) levels of mice with or without restraint stress at 0 and 9 h was detected by flow cytometry. Error bars show standard deviation; * *p* < 0.05, vs. 0 h groups; # *p* < 0.05, vs. no stress groups. n = 6 (three experiments with total six mice per group).

Tissue injuries can induce platelet activation, and such platelet responses are critical to initiate subsequent immune and repair processes [17,22]. Using a restraint stress mouse model, we analyzed the levels of circulating P-selectin expressing (P-selectin^hi^) platelets. Platelet counts and platelet surface CD41 do not change after stress (Figures S1 and S2). However, the levels of circulating P-selectin^hi^ platelets increased after the restraint stress (Figure 1), suggesting that stress-induced GI injury is associated with certain degrees of platelet activation. 

**Figure 2:**
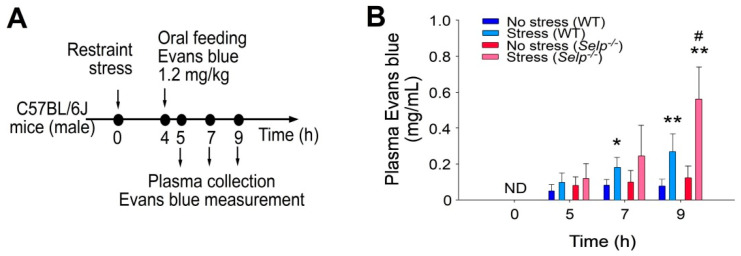
P-selectin deficiency exacerbates restraint stress-induced GI leakage in mice. (**A**) Experiment outline of restraint stress mouse model with Evans blue treatment. (**B**) Plasma Evans blue levels of wild-type (WT; C57BL/6J; *Selp^+/+^*; male) and P-selectin gene knockout (KO; *Selp^−^^/^^−^*; male) mice with or without restraint stress at 0, 5, 7, and 9 h. ND: not detected. Error bars shows standard deviation; * *p* < 0.05, ** *p* < 0.01, vs. respective no stress control groups; # *p* < 0.05, vs. WT groups. n = 6 (three experiments with total six mice per group).

P-selectin gene knockout (KO) (*Selp^−/^^−^*) mice were employed to specify the involvement of P-selectin regulation under stress. Using the restraint stress mouse model orally fed with Evans blue dye [15], we found that *Selp^−/^^−^* mice displayed higher GI leakage than the wild -type mice (Figure 2), implicating the protective role of P-selectin in the GI system. Because male (Figure 2) and female (Figure S3) mice behaved similarly in response to the stress, this suggested that there are no sex differences in stress-induced GI leakage in mice. 

### 2.2. Platelet-Transfer Ameliorated Stress-Induced GI Leakage and Inflammation, in which P-Selection Is a Critical Factor

**Figure 3:**
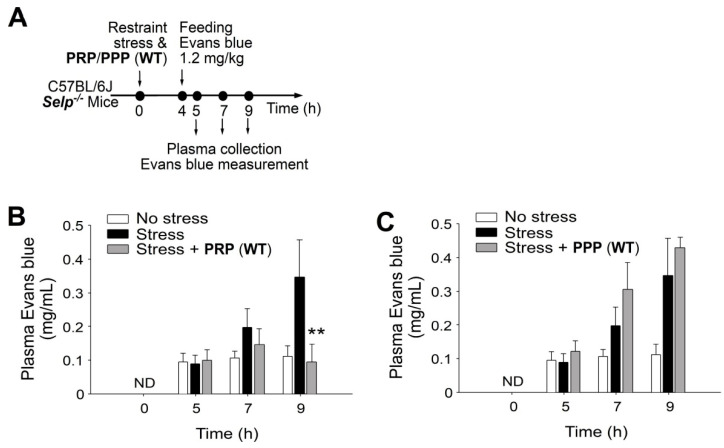
Wild-type (WT) mouse-derived platelet rich plasma (PRP) but not platelet-pool plasma (PPP) rescues stress-induced gut leakage in *Selp^−/−^* mice. (**A**) Experiment outline of restraint stress mouse model with Evans blue treatment. (**B**) Plasma Evans blue levels of *Selp^−/−^* mice without restraint stress (no stress) and with stress (stress) and stress plus PRP (WT) rescue (stress + PRP) at 0, 5, 7, and 9 h. ND: not detected. (**C**) Plasma Evans blue levels of *Selp^−/−^* mice without restraint stress (no stress) and with stress (stress) and stress plus PPP (WT) rescue (stress + PPP) at 0, 5, 7, 9 h. ND: not detected. ** *p* < 0.01, vs. respective stress groups. n = 6 (three experiments with total six mice per group).

**Figure 4:**
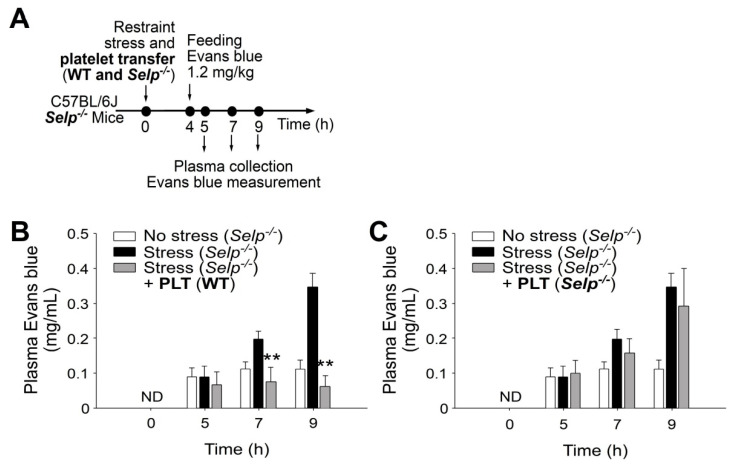
Transfer of wild-type (WT) washed platelets rescues stress-induced gut leakage in P-selectin-deficient (*Selp^−^^/^^−^*) mice. (**A**) Experiment outline of restraint stress mouse model with Evans blue treatment. (**B**) Plasma Evans blue levels of *Selp^−^^/^^−^* mice without restraint stress (no stress) and with stress [stress (*Selp^−^^/^^−^*)] and stressed *Selp^−^^/^^−^* mice transferred with wild-type platelets [stress (*Selp^−^^/^^−^*) + PLT (WT)] at 0, 5, 7, and 9 h. ND: not detected. (**C**) Plasma Evans blue levels of mice without restraint stress (no stress) and with stress (stress) and stressed wild-type mice transferred with platelets derived from *Selp^−^^/^^−^* mice [stress (*Selp^−^^/^^−^*) + PLT (*Selp^−^^/^^−^*)] at 0, 5, 7, and 9 h. ND: not detected. ** *p* < 0.01, vs. respective stress groups. n = 6 (three experiments with total six mice per group).

P-selectin is specifically expressed in platelets and endothelial cells [16,18]. If P-selectin expressing platelets play critical roles in the rescue of stress-induced GI injuries, then complementation by the transfer of platelet-rich plasma (PRP) from wild-type mice to *Selp^−/−^* mice could theoretically rescue restraint stress-induced GI leakage. The transfer using PRP, but not platelet-poor plasma (PPP; plasma without platelets) from the wild-type mice exerted a rescue effect (Figure 3A, experiment outline, 3B, 3C), suggesting the involvement of platelets in the rescue of stress-induced GI injuries. The purified platelets from wild-type and *Selp^−/−^* mice were transferred to *Selp^−/−^* mice to determine the rescue effect. Here, we found that platelets transferred from the wild type but not *Selp^−/−^* mice rescued GI leakage in *Selp^−/−^* mice (Figure 4). Because bleeding time of the recipient mice did not significantly change after platelet transfer, this indicated that the platelet transfer did not introduce obvious impacts on the coagulation system in the recipient mice (Figure S4).

**Figure 5:**
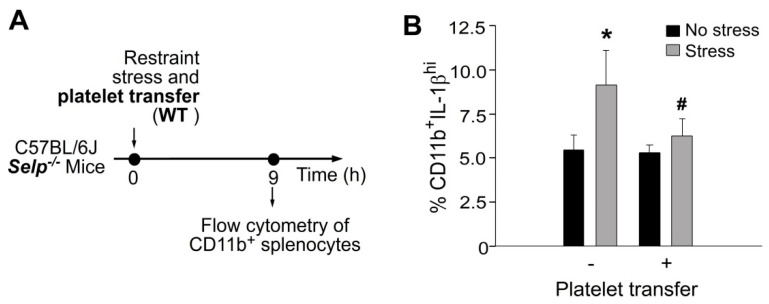
Stress-induced up-regulation of splenic CD11b^+^IL-1β^hi^ proinflammatory monocytes was ameliorated by platelet transfer in mice. (**A**) Experiment outline of restraint stress mouse model. (**B**) Flow cytometry analysis of CD11b^+^IL-1β^hi^ monocytes from *Selp^−/−^* mice with or without stress, and with or without wild-type (WT) platelet transfer. CD11b: monocyte marker; IL-1β: pro-inflammatory cytokine. * *p* < 0.05 vs. no stress groups; # *p* < 0.05 vs. without platelet transfer groups. n = 6 (three experiments with total six mice per group).

To investigate whether platelet transfer reduced stress-induced inflammation, percentage of splenic CD11b monocytes expressing high levels of interleukin-1β (IL-1β), a pro-inflammatory cytokine, was analyzed using flow cytometry (Figure 5). We found that complementation by the transfer of platelets from wild-type mice to *Selp^−/−^* mice markedly suppressed stress-induced high levels of CD11b^+^IL-1β^hi^ monocyte in *Selp^−/−^* mice (Figure 5). This suggests that wild-type platelet exerts an anti-inflammatory role in the *Selp^−/−^* recipients, in which P-selectin plays a critical role for platelet-mediated anti-inflammatory regulation.

### 2.3. Platelet-Mediated Protection Is Associated with Corrections of Stress-Induced Aberrant GI mRNA Expression

**Figure 6:**
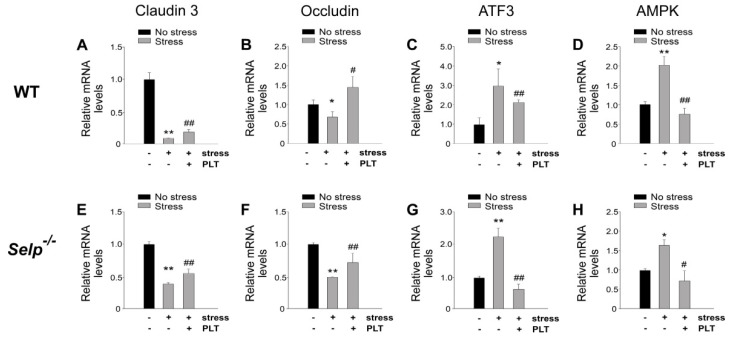
Relative mRNA expression levels of tight junction and stress-induced genes. Through qRT-PCR analysis, the relative mRNA expression levels of tight junction (CLDN3 and OLCN) (**A**,**B**,**E**,**F**) and stress-induced (ATF3 and AMPK) (**C**,**D**,**G**,**H**) genes, of wild type (WT; *Selp^+/+^*) (**A**–**D**) and P-selectin-deficient (*Selp^−^^/^^−^*) (**E**–**H**) mice in the duodenum with or without 9 h of restraint stress. The mRNA expression levels of control (no stress) groups were normalized to one-fold. * *p* < 0.05, ** *p* < 0.01, vs. respective no stress control groups. # *p* < 0.05, ## *p* < 0.01, vs. respective stress control groups without platelet transfer. n = 4 (two experiments with total four mice per group).

Stress-induced GI injuries are associated with the aberrant expression levels of GI tight junction mRNA [15,23]. Real-time quantitative polymerase chain reaction (qRT-PCR) analyses revealed that the mRNA expression levels of tight junction protein claudin 3 and occludin were down regulated, whereas stress-induced genes such as activating transcription factor 3 (ATF3) and AMP-activated protein kinase (AMPK) were up-regulated in mouse duodenal tissue after restraint stress (Figure 6). Whether platelet-mediated rescue is associated with the corrections of stress-induced aberrant GI mRNA expression was further analyzed. The aberrantly expressed GI mRNAs, including tight junctions claudin 3 (CLDN3) and occludin (OCLN) as well as stress-induced genes (ATF3 and AMPK) were re-balanced after the transfer of wild-type platelets into wild-type and *Selp^−/−^* mice (Figure 6).

### 2.4. Platelet-Mediated Protection Is Associated with Rescue of Stress-Mediated Suppression on GI Claudin 3 (CLDN3) Expression and Stress-Induced GI Epithelial Cell Apoptosis

**Figure 7:**
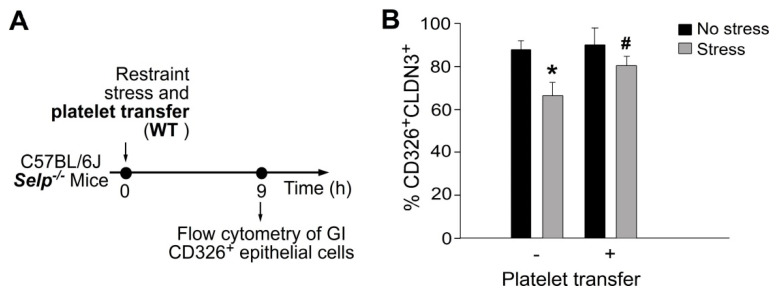
Stress-induced suppression of CLDN3 expression in GI CD326 epithelial cells was ameliorated by platelet transfer in mice. (**A**) Experiment outline of restraint stress mouse model. (**B**) Flow cytometry analysis of CD326^+^CLDN3^+^ epithelial cells from *Selp^−/−^* mice with or without stress, and with or without wild-type (WT) platelet transfer. CD326: epithelial cell marker; CLDN3: tight junction protein. * *p* < 0.05 vs. no stress groups; # *p* < 0.05 vs. without platelet transfer groups. n = 6 (three experiments with total six mice per group).

**Figure 8:**
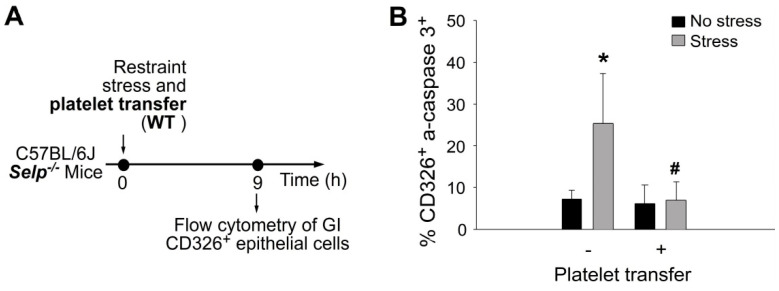
Stress-induced apoptosis of GI CD326^+^ epithelial cells are rescued by platelet transfer in mice. (**A**) Experiment outline of restraint stress mouse model. (**B**) Flow cytometry analysis of GI CD326^+^ epithelial cells from *Selp^−^^/^^−^* mice with or without stress, and with or wild-type (WT) platelet transfer. CD326: epithelial cell marker; active-form caspase 3 (a-caspase 3): apoptotic cell marker. * *p* < 0.05 vs. no stress groups; # *p* < 0.05 vs. without platelet transfer groups. n = 6 (three experiments with total six mice per group).

Restraint stress can suppress the expression of GI epithelial tight junction and further induce apoptosis of GI epithelial cells [15]. A complementation experiment was performed by transferring wild-type mouse platelets to *Selp^−/−^* recipient mice to investigate whether platelet transfer can rescue stress-mediated suppression on tight-junction expression and stress-induced apoptosis in GI epithelial cells. In agreement with the rescue effect of platelet transfer in Evans blue assay (Figure 2) and mRNA expression (Figure 5), the complementation experiment indicated that the transfer of wild-type mouse platelets ameliorated restraint stress-induced suppression (Figure 7) and suppressed stress-induced enhancement of CD326^+^ active-form caspase 3^+^ double-positive GI epithelial cells in the *Selp^−/−^* recipients (Figure 8). 

## 3. Discussion

Platelet has a long history of clinical use for patients with thrombocytopenia and bleeding disorders to restore hemostasis and enhance tissue repair [24,25]. Purified platelets and PRP are tissue regenerative agents; injections of a concentration of a patient’s own platelets can accelerate the healing of injured tendons, ligaments, muscles and joints [17,24,26,27,28]. In GI tissue repair, PRP exerts beneficial effects in intestinal anastomosis animal models [29,30,31,32]. At the same time, PRP accelerated epithelialization and healing in patients with chronic anal fissures [33]. However, GI surgery and local treatments of PRP are needed in these studies. Here, we found that intravenous injection of PRP and purified platelets is a feasible approach, sufficiently rescued stress-induced GI injury without invasive operations.

Platelets expressed a variety of growth factors such as platelet-derived growth factor, vascular endothelial growth factor, transforming growth factor-β, fibroblast growth factor, insulin-like growth factor, and hepatocyte growth factor [28,34]. These growth factors can help healing and regeneration of damaged tissues, accelerated neovascularization, increasing blood flow and nutrition in wound-surrounding cells [28,34]. Therefore, the tissue repairing effects of PRP, and purified platelets are likely attributed to these platelet-associated factors. 

Despite that various platelet-derived factors have been implicated in the tissue repair effect, the mechanism of platelet-mediated tissue repair and cell pro-survival effect remains largely unknown. In addition, the critical platelet factor that contributes to the pro-survival effect remains elusive. The present report suggests that P-selectin is one of the key pro-survival platelet factors, because P-selectin deficiency results in insufficient rescue of platelets against stress-induced GI injury. P-selectin is an adhesion molecule expressed on the surfaces of activated platelets [16,18]. This suggests that cellular interaction between platelets and injured target cells is a critical step in the rescue of stress-induced GI cell injury. Theoretically, such platelet-target cell interaction and P-selectin-down-stream signals may facilitate the release of platelet-associated growth factor, and thus conducts pro-survival cellular regulation. Meanwhile, transferred platelets also exert anti-inflammatory properties [35,36]. This raises another possibility that, in addition to GI epithelial cells, leukocytes are also target cells of transferred platelets. In agreement with this, here we found that platelet transfer considerably reduced stress-induced up-regulation of pro-inflammatory CD11b^+^IL-1β^hi^ monocytes in the spleen of *Selp^−^^/^^−^* mice (Figure 5). Because P-selectin plays critical role in leukocyte-platelet interaction during inflammation [37,38], P-selectin^+^ platelets may ameliorate stress-induced inflammation thereby facilitate the subsequent tissue repair. Because the detailed mechanism remains elusive, P-selectin-dependent platelet-mediated rescue in the tissue repair and anti-inflammation regulation are worthy of further investigations.

A growing body of evidence suggests that the soluble form P-selectin exerts anti-inflammatory and cell pro-survival roles [19,20,21,39]. Although membrane-form P-selectin is associated with inflammation progression [40], anti-inflammatory soluble P-selectin can be released from the surfaces of platelets and endothelial cells in the forms of soluble ectodomain and cell-extracellular vesicle associated protein [20,41]. Treatments of soluble P-selectin can mobilize anti-inflammatory CD34^+^ stem cells and block leukocyte infiltration -mediated inflammation [19,20,21,39], suggesting the anti-inflammatory properties of soluble P-selectin. Hence, P-selectin exerts dual roles in inflammatory regulation depending on expression type (soluble vs. cell-surface bound) and tissue environment.

In this study, we found that the transfer of wild-type platelets can rescue stress-induced GI leakage in *Selp^−^^/^^−^* mice. The stress-induced apoptosis of CD326^+^ GI epithelial cells was rescued by the transfer of wild type, but not P-selectin-deficient platelets. These results collectively suggest that platelet is a critical cell type for maintaining the GI homeostasis under stress, and that P-selectin could be a potential molecular target for managing stress-induced GI tract injury.

## 4. Materials and Methods

### 4.1. Laboratory Mice 

Wild-type C57BL/6J mice aged 8–12 weeks were purchased from the National Laboratory Animal Center (Taipei, Taiwan) [42,43,44,45,46,47]. Genetically deficient *Selp*^−*/*−^ (B6; 129S2-*Selp^tm1Hyn^*/J; P-selectin KO) [19,21,48] with a C57BL/6J background were obtained from the Jackson Laboratory (Maine, USA). *Selp*^−*/*−^ mice were backcrossed with WT C57Bl/6J mice over six generations. The genotype of the P-selectin KO mice was routinely checked every 15-20 generations following the protocols provided by the Jackson Laboratory (https://www.jax.org/Protocol?stockNumber=002217&protocolID=23572) (accessed on 1 July 2022). The phenotype (lack of platelet P-selectin expression) of the P-selectin KO mice was routinely checked every generation using flow cytometry analysis (Figure S5, *Selp*^−*/*−^ groups). All animals were housed in the Animal Center of Tzu-Chi University in a specific pathogen-free, light- and temperature-controlled environment with free access to food and filtered water. Approximately 360 wild-type mice and 100 *Selp*^−*/*−^ mice were employed. All protocols for examining the experimental animals were approved by the Animal Care and Use Committee of Tzu-Chi University, Hualien, Taiwan (approval ID: 110024). 

### 4.2. Induction and Measurement of Stress-Induced GI Leakage

A mouse model of restraint-stress GI leakage fed with Evans blue dye was established based on previously described methods [15]. Sex (male and female), age (10–14 weeks old) and body weight (>25 g) matched mice were kept in a 50-mL plastic falcon tube for 9 h to induce restraint stress [15,49,50]. Holes were created at the tapering end of the Falcon tube to ensure sufficient air supply. Blood samples (50 µL) were collected during the experiment at 0, 5, 7 and 9 h, after the stress challenge. Evans blue (1.2 g/kg, Santa Cruz Biotechnology, Santa Cruz, CA, USA) was fed to the mice using a steel feeding tube 4 h after the commencement of stress challenge [15]. Blood plasma was isolated by collecting blood in an Eppendorf tube and mixing it with an equal proportion of anti-coagulant citrate dextrose solution to prevent coagulation [51,52,53]. The collected plasma was transferred to 96 well plates, in which the concentration of Evans blue was determined using a full-spectrum analyzer (Multiskan Spectrum, Thermo Fisher Scientific, Waltham, MA, USA) at 620 nm. 

### 4.3. Platelet, PRP and PPP Transfer

Platelet counts of mice were measured using a hematology analyzer (KX-21N; Sysmex, Kobe, Japan) [48,53,54]. Platelet and PRP purification [53,55], and adopted platelet transfer [56] were performed based on the protocols described previously. The recipient mice were not subjected to a platelet depletion preparation before the transfer of platelet, PRP and PPP. For PRP preparation, 1mL of whole blood was collected from the mice and added with 1:4 anticoagulant acid–citrate–dextrose (ACD) solution (38 mM citric acid, 75 mM sodium citrate, 100 mM dextrose) [52,57] and 500 μL of Tyrode’s buffer (137 mM NaCl, 2.8 mM KCl, 2 mM MgCl_2_, 0.33 mM NaH_2_PO_4_, 5 mM dextrose, 0.35% bovine serum albumin; 10 mM HEPES, pH 7.4) [54]. The samples were centrifuged at 180× *g* for 10 min. The colloidal supernatant was transferred to a clean Eppendorf tube and injected (300 μL) intravenously into each mouse [55]. For platelet purification, the whole blood from the mice was mixed with 6:4 ACD and 500 μL Tyrode’s buffer and centrifuged at 160× *g* for 5 min. The colloidal layer was then transferred to a clean Eppendorf tube, added with addition of 10 mM ethylene diamine tetraacetic acid, and centrifuged again at 300× *g* for 5 min. The pellet was then resuspended in Tyrode’s buffer and injected (300 μL containing approximately 35 × 10^9^/L platelets) intravenously to each mouse. 

### 4.4. Bleeding Time Experiment

Following previous described methods [53,58], bleeding time experiments were performed. Mice with or without stress were anesthetized (intraperitoneal injection of ketamine: xylazine, 80: 10 mg/kg body weight [59]) before bleeding time analysis. The tails of mice were prewarmed for 5 min at 37 °C in a water bath, and a 5 mm segment of tail tip was amputated with a razor blade. The bleeding time was measured until the bleeding stopped [58].

### 4.5. qRT-PCR Analysis

#### 4.5.1. RNA Isolation and cDNA Preparation

RNA isolation and cDNA preparation were performed based on previously described methods [15]. Mouse duodenum samples (1 cm; begins immediately after the pyloric portion of the stomach) were isolated, washed (phosphate-buffered saline, PBS) and dissolved in Trizol (Ambion, Thermo Fisher Scientific) after 9 h of stress challenge. After standard isolation protocols, RNA concentration was analyzed using Nano drop spectrophotometer (Thermo Fisher Scientific). The isolate RNA (1 μg) was used to synthesize complementary DNA (cDNA) on an iScript cDNA Synthesis Kit (Bio-Rad Laboratories, Hercules, CA, USA). The obtained cDNA was used for PCR and qRT-PCR analyses and the samples were stored at −20 °C before use [15].

#### 4.5.2. qRT-PCR

We used qRT-PCR to analyze the expression of tight junction genes, such as CLDN3, OCLN, ATF3 and AMPK, involved in the maintenance of mucosal homeostasis and intestinal integrality [15,60,61,62,63], in GI tissues. cDNA (2 µL) was mixed with 10 µL of SYBR Green (Thermo Fisher Scientific), 0.5 µL each of forward and reverse primers, and 7 µL of ddH_2_O. cDNA was then quantified on real-time reverse transcription linkage instrument (StepOnePlus Real-Time PCR System, Thermo Fisher Scientific) with varying annealing temperatures according to the primers. Each sample was run in triplicate and the average cycles threshold (Ct) values were used to calculate 2^−ΔΔCT^ withGAPDH (glyceraldehyde-3-phosphate dehydrogenase) gene expression as internal control. The primer sequences are listed in Supplementary Table S1.

### 4.6. Flow Cytometry Analysis

Flow cytometers (FACScalibur, BD Biosciences and Gallios, Beckman Coulter Life Sciences) [15,19,21] were used for analyzing the expression levels of platelet surface P-selectin and CD41 (integrin α_IIb_) before and after stress. Platelet surface P-selectin and CD41 were stained by anti-mouse phycoerythrin-conjugated P-selectin Ig (eBioscience, Thermo Fisher Scientific) [53] and Alexa Fluor 488-conjugated anti-mouse CD41 Ig (clone MWReg30 [54]; Biolegend, San Diego, CA, USA), respectively. Mouse GI epithelial cells were analyzed following described methods [15]. Mouse duodenum samples (6 cm, begins immediately after the pyloric portion of the stomach; PBS washed) were cut into tiny pieces and incubated with a collagenase D (Sigma-Aldrich, Sigma-Aldrich, Burlington, MA, USA; 1 mg/mL)-containing serum-free cell culture medium for 30 min in a 15 mL Falcon tube at 37 °C with shaking (upright shaking incubator; OSI 500, Kansin Instruments, New Taipei City, Taiwan) after 9 h of restraint stress. Mouse GI epithelial cells were dissociated from the remaining cell clusters and tissue pellets by incubation with 2 mL of non-enzymatic cell-dissociation solution (Sigma-Aldrich) for 10 min at 25 °C. After being washed, each sample of the dissociated cells was fixed with 500 µL of a fixation buffer (Cytofix, BD Biosciences, San Jose, CA, USA), mixed properly and incubated at 25 °C for 20 min. The samples were then centrifuged at 300× *g* for 5 min. After washing (Perm/Wash buffer, BD Biosciences) and blocking (5% bovine serum albumin in RPMI), the cells were subjected to staining of CD326 (a gut epithelial cell marker [64,65]; anti-CD326 antibody, BioLegend), CLDN3 (tight junction; anti-CLDN3 antibody, Thermo Fisher Scientific) and cell-death marker (apoptosis; anti-cleaved caspase-3 antibody, Cell Signaling Technology) [15]. For monocyte analysis, mouse splenocytes were isolated as previously described [54]. Levels of mouse CD11b^+^IL-1β^hi^ (anti-CD11b antibody, Thermo Fisher Scientific [66,67]; anti-IL-1β antibody, BioLegend) pro-inflammatory splenic monocytes were analyzed by flow cytometry before (0 h) and after (9 h) restraint stress. The samples were subjected to flow cytometry (Gallios, Beckman Coulter Life Sciences) for quantitative analysis as previously described [15].

### 4.7. Statistical Analyses

The experimental results were analyzed using Microsoft Office Excel 2003 and SPSS 17 and reported as mean ± standard deviation. Statistical significance of the obtained results was examined using one-way analysis of variance and post hoc Bonferroni-corrected *t* test. A probability of type 1 error α = 0.05 was considered the threshold of statistical significance. 

## Data Availability

Not applicable.

## Supplementary Materials

The following supporting information can be downloaded at: https://www.mdpi.com/article/10.3390/ijms231911909/s1.
