# Supplementary material for: P-Selectin is a Critical Factor for Platelet-Mediated Protection on Restraint Stress-Induced Gastrointestinal Injury in Mice
Source: Int J Mol Sci. 2022 Oct 7;23(19):11909. doi: 10.3390/ijms231911909 (PMC9570147; doi:10.3390/ijms231911909)
Supplement: Supplementary file 1 [file ijms-23-11909-s001.zip › ijms-1906046-supplementary.pdf]

International Journal of *Molecular Sciences*

MDPI

Supplementary information

P-selectin is a critical factor for platelet-mediated protection on restraint stress-induced gastrointestinal injury in mice

Subhashree Pethaperumal[1], Shih-Che Hung[2], Te-Sheng Lien[1], Der-Shan Sun[1,2], and Hsin-Hou Chang,[1,2*]

[1] Department of Molecular Biology and Human Genetics, Tzu-Chi University, Hualien 970, Taiwan. SP
[2] Institute of Medical Sciences, Tzu-Chi University, Hualien, 970, Taiwan. SCH
* Correspondence: HHC, hhchang@mail.tcu.edu.tw.

Running title: P-selectin expressed platelet protects gut injury

Key words: Platelet transfer, restraint stress, gastrointestinal injury, gastrointestinal leakage, gastrointestinal epithelial cell apoptosis, P-selectin

* To whom correspondence and reprint requests should be addressed.

Hsin-Hou Chang Ph.D. Room D407, Tzu-Chi University, No. 701, Section 3, Chung-Yang Road, Hualien 97004, Taiwan. Tel: 886-3-8565301 ext 2667. Fax: 886-3-8578386. E-mail: hhchang@mail.tcu.edu.tw

Figures S1-S5: pages 2-6

Table S1: page 7

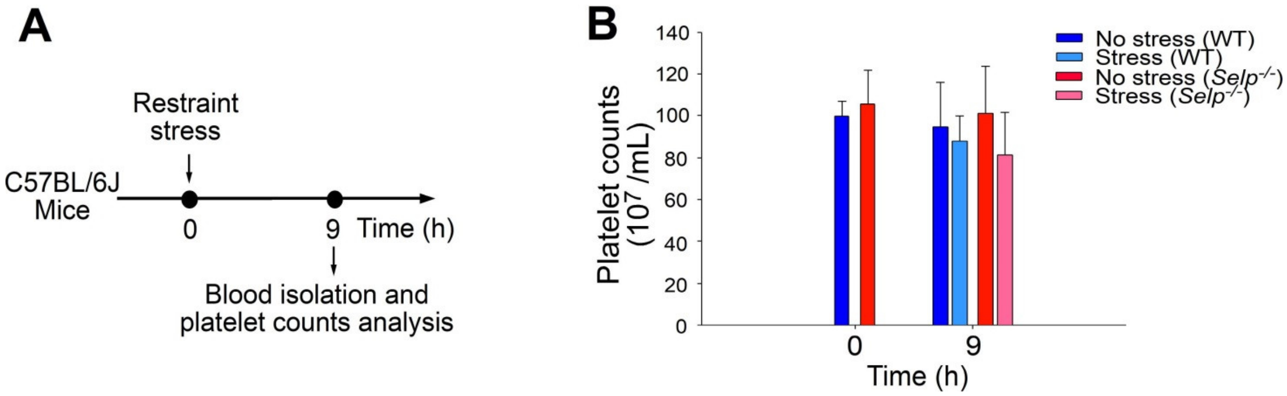

Figure S1. Platelet counts of wild type (WT; *Selp*$^{+/+}$) and P-selectin deficient (*Selp*$^{-/-}$) mice were analyzed before (0h) and after (9h) restraint stress. (A) Experiment outline of restraint stress mouse model and platelet count analysis. (B) Analysis of mouse platelet counts. Error bars show standard deviation. n = 6 (3 experiments with total 6 mice per group).

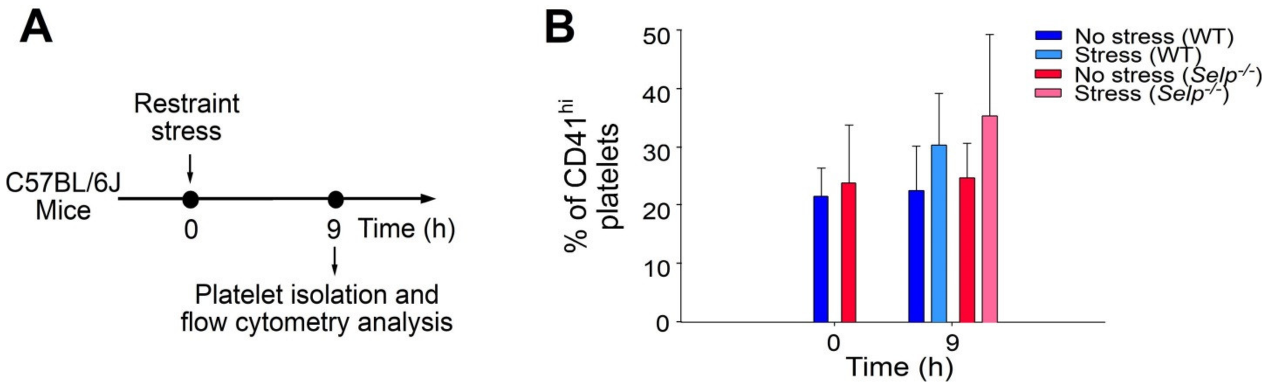

Figure S2. Percentage of circulating CD41hi platelets was not statistically significant changed after 9 hour restraint stress in mice. (A) Experiment outline of restraint stress mouse model and platelet analysis. (B) Percentage of platelets expressing high levels of surface CD41 (integrin α_{IIb}) (CD41hi) in mice with or without restraint stress at 0 and 9 h was detected by flow cytometry. Error bars show standard deviation. n = 6 (3 experiments with total 6 mice per group).

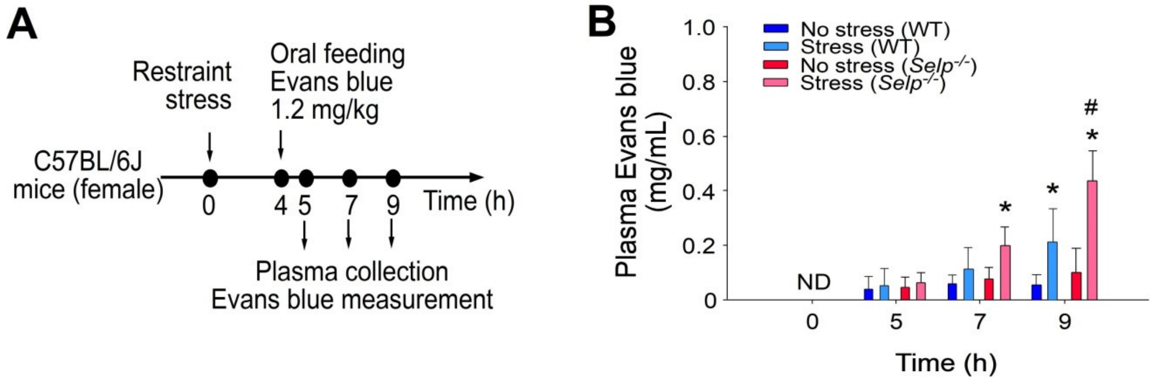

Figure S3. P-selectin deficiency exacerbates restraint stress-induced GI leakage in female mice. (A) Experiment outline of restraint stress mouse model with Evans blue treatment. (B) Plasma Evans blue levels of wild-type (WT; C57BL/6J; *Selp*$^{+/+}$; female) and P-selectin gene knockout (KO; *Selp*$^{-/-}$; female) mice with or without restraint stress at 0, 5, 7, and 9 h. ND: not detected. Error bars shows standard deviation; * $P < 0.05$, ** $P < 0.01$, vs. respective no stress control groups; # $P < 0.05$, vs. WT groups. n = 6 (three experiments with total six mice per group).

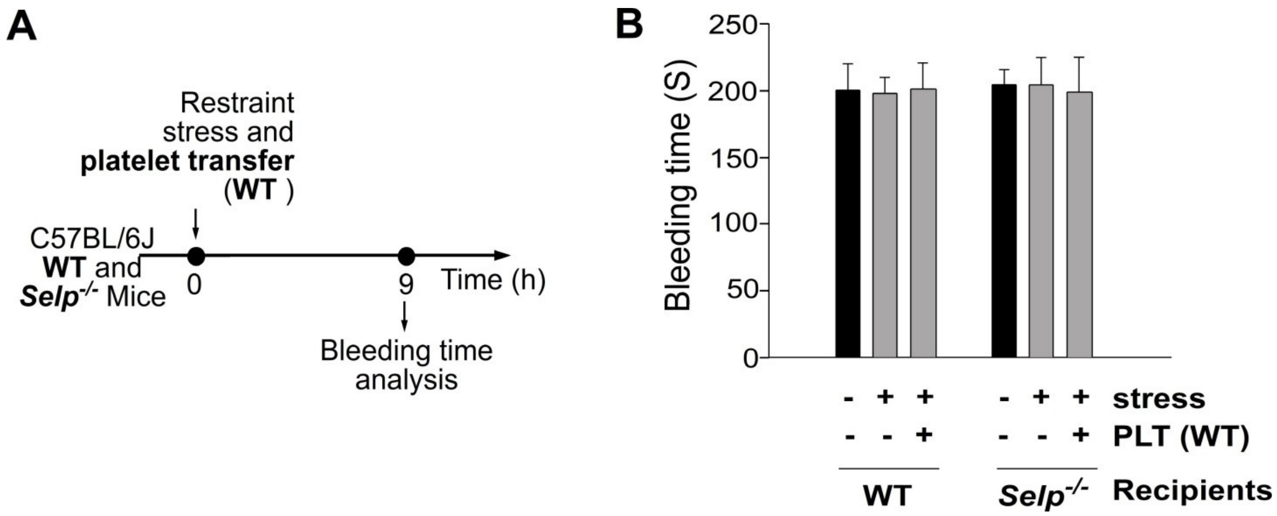

Figure S4. Bleeding time analysis. Bleeding time was analyzed in wild type (WT) and *Selp*$^{-/-}$ mice with (9 h) or without (0 h) stress, and with or without wild-type (WT) platelet transfer. n = 6 (three experiments with total six mice per group).

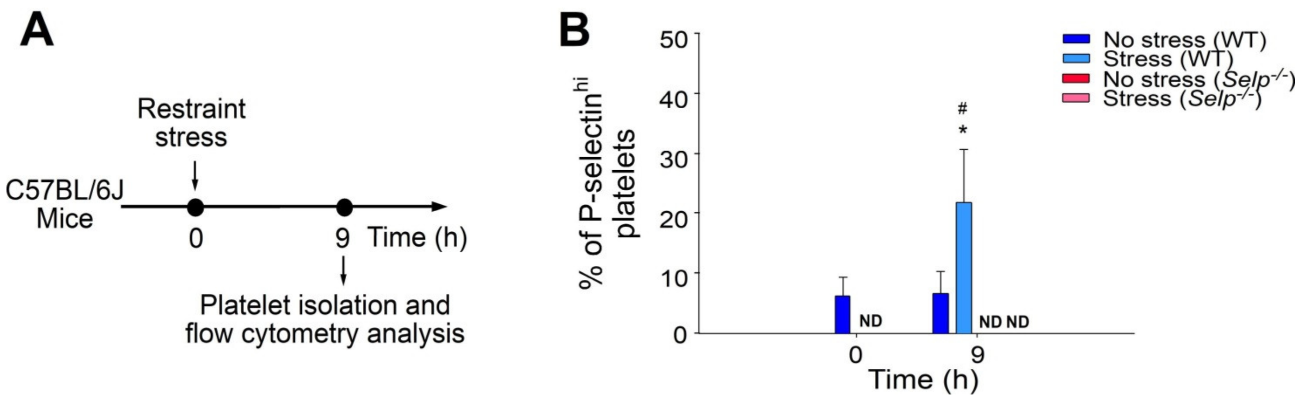

Figure S5. Percentage of circulating P-selectin^{hi} platelets was up-regulated after 9 hour restraint stress in mice. (A) Experiment outline of restraint stress mouse model and platelet analysis. (B) Percentage of platelets expressing high surface P-selectin (P-selectin^{hi}) levels of mice with or without restraint stress at 0 and 9 h was detected by flow cytometry. The platelet surface P-selectin expression only detected in wild type (WT; *Selp*$^{+/+}$) but not in P-selectin deficient (*Selp*$^{-/-}$) mice. ND: not detected. Error bars show standard deviation; * $P < 0.05$, vs. 0 h groups; # $P < 0.05$, vs. no stress groups. n = 6 (3 experiments with total 6 mice per group).

Table S1. List of primers used in this report.

Gene symbol	Forward primer (5′→3′)	Reverse primer (5′→3′)	Product size (bp)	Temperatur e (°C)
**GAPDH**	TCAACAGCAAC TCCCACTCTTC CA	ACCCTGTTGC TGTAGCCGTA TTCA	115	55
**Occludin**	TCTTGGGAGCC TGGACATTT	TGCATCTCTCC GCCATACAT	159	65
**CLDN3**	CCACTACCAGC AGTCGATGA	GGAAGGGCG AGGTTTCTTTG	211	60
**ATF3**	AGTGACAGCAT GAGCCCTCT	GCAGCACTGA CCTGATCAAA	179	60
**PRKAA1 (AMPK)**	GAAACCTGAG AACGTCCTGC	GCCTGCGTAC AATCTTCCTG	157	54
